# Strong Association Between Placental Pathology and Second-trimester Miscarriage

**Published:** 2021

**Authors:** H J Odendaal

**Affiliations:** Department of Obstetrics and Gynaecology, Faculty of Medicine and Health Sciences, Stellenbosch University, Tygerberg, P O Box 241, Cape Town 8000, South Africa

**Keywords:** Autopsy, Placental histology, Second-trimester miscarriage

The survival probability of early human conceptions is very low. At least 73% of natural single conceptions have no real chance of surviving six weeks of gestation [1]. After six weeks, survival rates improve rapidly as 90% of the remainder will survive to term. This low fetal loss rate is close to the low rates of 1% - 2.9% for different methods of artificial reproduction [2]. From 16 weeks the rate of loss reduces further, to around 1% [3,4]. Miscarriage is the loss of pregnancy before fetal viability and has a pooled risk of 15.3% (95% CI: 12.5–18.7) [5]. The population prevalence of women who have had one miscarriage is 10.8% (95% CI: 10.3 −11.4). As there is a great need for better knowledge and services, a recent editorial article in The Lancet pleaded for worldwide reform to improve the care of women who have had a miscarriage [6].

Very little information is available on rates and common causes of spontaneous second- or mid-trimester miscarriage (also referred to as late abortion), possibly due to the global lack of reporting systems. The National Vital Statistics System in the United States reports fetal mortality from 20 week’s gestation [7]. In the UK, the cut-off point for viability is 24 weeks [8], while 22 weeks is recommended by the World Health Organization [9] and in Europe [10]. While national statistics of these fetal deaths are kept, there is no information about losses that occur before the mentioned cut-off gestation periods.

In the UK, 12–24 weeks gestational age is defined as a late miscarriage [11,12] with prevalence rates varying between 0.7% and 3%. Simpson et al. [3] showed a spontaneous miscarriage rate of 0.4% between 15 and 21 weeks in 264,653 pregnancies. Other publications report the rates of second-trimester miscarriages as between 0.72%−2.9% of pregnancies [11–13].

## Placental Inflammation

The first publications on the possible causes of second-trimester miscarriage emphasised the role of placental inflammation. A retrospective study by Russel [14], examining the placental histology of 7,705 spontaneous deliveries for the presence of histological chorioamnionitis, found that for deliveries between 21 and 24 weeks, the prevalence was 94.4%. In contrast, histological chorioamnionitis was present in 40% and 10.7 % for deliveries at 25–28 weeks and 33–36 weeks, respectively.

A study of 360 spontaneous abortions showed placental inflammatory lesions in a third of cases of early fetal death at 13–18 weeks gestation but in 60% when the fetal death was at 18–24 weeks [15]. Another study of 656 consecutive couples who had at least two spontaneous miscarriages and referred to a dedicated miscarriage clinic, 158 had a miscarriage in the second-trimester [16]. Possible underlying causes were idiopathic (50%), antiphospholipid antibody syndrome (33%), cervical weakness (8%), uterine anomaly (4%), infection (3%), and hypothyroidism (2%). Heller et al. [17] reviewed medical records and pathologic material of spontaneous second-trimester pregnancy losses. From the 67 cases available, 38 cases (56.7%) showed histologic acute chorioamnionitis. In another study on 118 second-trimester abortions, stillbirths, and perinatal deaths, placental findings showed that infection occurred most frequently in abortions (58.2%), the most frequent cause of death in this group [18]. Finally, a review by Ugwumadu [19] concluded that chorioamnionitis is the commonest cause of mid-trimester pregnancy miscarriages and extreme preterm delivery.

## Other Common Causes

The placenta plays a key role in implantation and development of the fetus. Soluble Fms-like kinase and placental growth factor are both involved in placental vascularization. Andersen et al. [20] examined the serum of 1,676 pregnant women, donated during early pregnancy (before a gestation of 22 weeks), for soluble Fms-like kinase and placental growth factor. Spontaneous abortion occurred in 59 cases (3.5%). Continuous increased concentrations of both Fms-like kinase and placental growth factor were significantly associated with decreased hazard ratio for spontaneous abortion [20].

Factor V and the prothrombin mutations have been associated with an approximate tripling of the risk for late miscarriages [21]. The authors postulated that placental thrombosis may be the underlying pathogenic mechanism. Another probable cause for mid-trimester abortions may be the presence of antiphospholipid antibodies which was found in 26.5% of women (n = 200) with recurrent mid-trimester abortions (Table 1) [22].

## Defective Placentation

Defective haemochorial placentation as a cause of miscarriage was first reported in 1987 [23]. None of the normal vascular adaptive changes were observed in any of the second-trimester miscarriages (n = 5). The authors stated that their preliminary results, not previously described, strongly supported the concept that miscarriages and pregnancies, complicated by preeclampsia and fetal growth restriction, may be a continuum of disorders with similar pathology in the placental bed.

In a study on spiral artery transformation and trophoblast invasion, 96 spiral arteries in 26 pregnancies at 13 weeks or later were compared to 236 spiral arteries in 74 normal pregnancies matched for gestational age [24]. Compared with normal pregnancy, myometrial spiral arteries in late miscarriage showed significantly reduced endovascular and intramural trophoblast, and less extensive fibrinoid change. In contrast, endovascular trophoblast in decidual spiral arteries was significantly increased. As found in preeclampsia, late spontaneous miscarriage was associated with reduced trophoblast invasion and inadequate transformation of myometrial spiral arteries. These findings on the placental bed are in line with current concepts that ‘great obstetrical syndromes’, which includes late abortion, are associated with disorders of deep placentation [25,26].

Valuable information on possible causes of late miscarriage came from the Safe Passage Study, a prospective investigation on the effects of alcohol exposure during pregnancy on stillbirths and sudden infant deaths [27]. When a stillbirth had occurred at 20 week’s gestation or beyond, the mother was approached for consent for autopsy [28]. Gestational ages were confirmed during autopsies [29], where in 14 of the pregnancies, the gestational age was less than 20 weeks. This afforded the opportunity to investigate autopsy reports in 13 fetuses of whom 12 had histological examination of the placenta. Examination of the placenta and all autopsies were done according to a standard protocol [30]. The most prevalent histological abnormalities were placental abruption, seen in six second-trimester miscarriages, occasionally on its own or in combination with acute chorioamnionitis or maternal vascular malperfusion (MVM) (Table 2). The second most frequent finding was MVM, also as a single finding or in combination with others. The third most frequent pathology was acute chorioamnionitis, in combination or alone. Acute chorioamnionitis, as a single diagnosis occurred only once. Other causes for the miscarriages included diffuse chronic villitis due to cytomegalovirus infection and early amnion rupture with anhydramnios and cord obstruction. Other abnormalities were rarely seen.

From the abovementioned studies, it is clear that placental pathology plays a major role in the etiology of spontaneous second-trimester miscarriages. Causes of fetal demise at the end of the second-trimester differ little from that of stillbirths. Therefore, placental histology in second-trimester miscarriages may be of great help to identify risks in future pregnancies as the same complications tend to repeat. Interestingly, placental disease was also the leading cause of antepartum stillbirths (26%) in the 633 stillbirths examined by the Stillbirth Network. However, they did not differentiate between the different placental conditions such as MVM or acute chorioamnionitis [31]. In the Dutch Cohort of 750 antepartum stillbirths, 65% were due to placental disease but intrauterine infection was found in only 1.8% of stillbirths [32].

As some of the causes of second-trimester miscarriages and early stillbirths are similar, information on the causes and rates of miscarriage may provide valuable information for efforts to reduce stillbirths. It is necessary to analyse stillbirth and perinatal mortality rates regularly to project whether the millennium goals for 2030 will be reached [4]. Although there has been a 25.5% decline in global stillbirth rates from 2000 to 2016, the slow improvement in sub-Saharan Africa is a cause for concern [33]. Every opportunity should therefore be used to obtain additional information regarding stillbirths. As conditions related to poor placentation tend to repeat in subsequent pregnancies, histologic findings suspicious of placental insufficiency could alert one to provide specific care. Much can be done such as determining flow velocity waveforms for screening for fetal growth restriction [34] and prophylactic administration of aspirin to women at risk for preeclampsia [35] or early delivery of women with preeclampsia [36].

## Predisposing Conditions

It is important to keep in mind that there are several predisposing conditions to miscarriages including genetic, anatomical, endocrine, immunological, infective, and environmental.

## Future Reform

**Figure 1:**
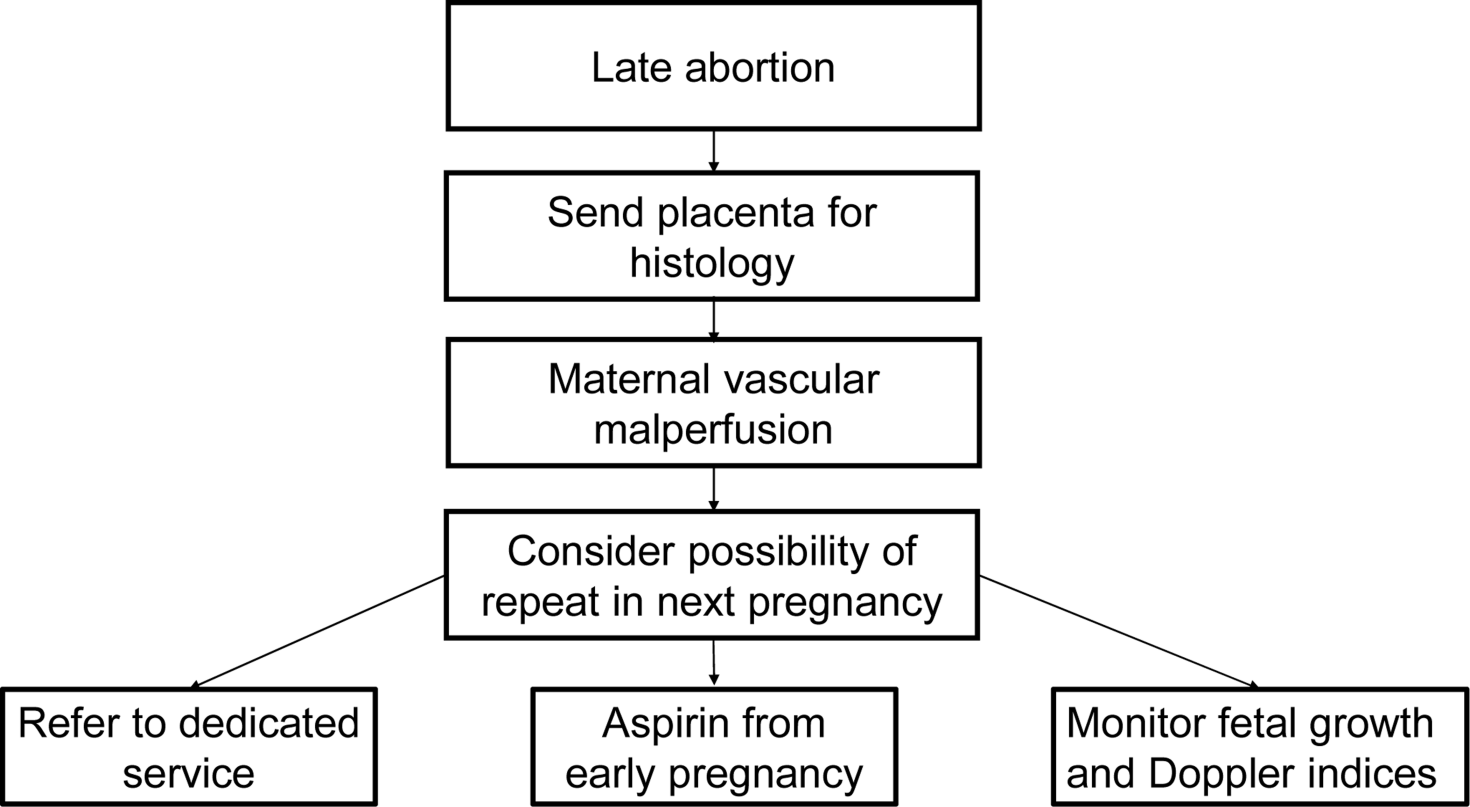
One of the ways how knowledge of the cause of a second-trimester miscarriage could help to manage subsequent pregnancies.

As stated earlier, worldwide reform is needed in the care of women after they have had a miscarriage [6]. Part of the reform should be more attention to second-trimester miscarriages, which is now considered as part of placental bed disorders including preterm labor, preterm rupture of membranes, abruptio placentae, fetal growth restriction, and preeclampsia [37]. National statics, similar to that of stillbirths, should be kept for second-trimester miscarriages and placental histology, if feasible, should be requested in all cases of spontaneous mid-trimester miscarriage as knowledge about the cause of the miscarriage would help to better manage future pregnancies (Figure 1).

## Figures and Tables

**Table 1:** Common natural causes of late second-trimester miscarriages (demise before 22 weeks’ gestation).

Uterine anomaly
Cervical weakness
Antiphospholipid antibodies
Infection
Defective placentation

**Table 2:** Placental histology demonstrating the probable cause of death in late second-trimester miscarriages.

Gestational age at birth		Birth weight (g)	Placental weight (g)	Histological findings
**According to early ultrasound**	**According to femur length**			
21w2d	21w6d	408	154	Acute chorioamnionitis, abruption
22w0d	16w3d	60	42	Maternal vascular malperfusion
22w6d	16w6d	160	140	Chronic villitis with obliterative fetal vasculopathy, cytomegalovirus
24w4d	17w5d	148	70	Early amnion rupture with anhydramnios, cord obstruction
21w3d	14w3d	50	41	Maternal vascular malperfusion
21w1d	23w1d	450	N/A	Not done
21w6d	22w6d	410	104	Acute chorioamnionitis, abruption
20w6d	20w2d	331*	82	Acute chorioamnionitis
20w6d	14w4d	103*	49	Termination of pregnancy for Turner syndrome
19w3d	N/A	N/A	282	Acute chorioamnionitis, abruption, maternal vascular malperfusion
21w2d	22w1d	336*	132	Abruption
22w3d	15w2d	62*	31	Maternal vascular malperfusion, abruption
21w1d	16w3d	71*	N/A	Not done
21w4d	22w4d	400	131	Maternal vascular malperfusion, abruption

* No birth weight available, only autopsy weight

Adapted from Odendaal et al. [38]
